# Reiber’s Diagram for Kappa Free Light Chains: The New Standard for Assessing Intrathecal Synthesis?

**DOI:** 10.3390/diagnostics9040194

**Published:** 2019-11-16

**Authors:** Philipp Schwenkenbecher, Franz Felix Konen, Ulrich Wurster, Torsten Witte, Stefan Gingele, Kurt-Wolfram Sühs, Martin Stangel, Thomas Skripuletz

**Affiliations:** 1 Department of Neurology, Hannover Medical School, Carl-Neuberg-Str. 1, 30625 Hanover, Germany; Schwenkenbecher.Philipp@mh-hannover.de (P.S.); Konen.Felix@mh-hannover.de (F.F.K.); Wurster.Ulrich@mh-hannover.de (U.W.); Gingele.Stefan@mh-hannover.de (S.G.); Suehs.Kurt-Wolfram@mh-hannover.de (K.-W.S.); Stangel.Martin@mh-hannover.de (M.S.); 2 Department of Clinical Immunology & Rheumatology, Hannover Medical School, Carl-Neuberg-Str. 1, 30625 Hanover, Germany; Witte.Torsten@mh-hannover.de

**Keywords:** cerebrospinal fluid, Reiber’s diagram, kappa free light chains, hyperbolic function, biomarker, multiple sclerosis

## Abstract

Oligoclonal bands are the gold standard for determination of an intrathecal immunoglobulin G synthesis and were recently included in the McDonald criteria of 2017 to diagnose relapsing multiple sclerosis (MS) as a substitute for dissemination in time. Intrathecally produced kappa free light chains (KFLC) are a novel promising biomarker with similar characteristics and the advantage for automated determination. However, different approaches exist to determine the intrathecal KFLC fraction. The most common method is to calculate the CSF/serum KFLC quotient with reference to the albumin CSF/serum quotient (Q_Kappa_/Q_Alb_) the so-called KFLC index. Recently, Reiber developed a theoretically and empirically founded hyperbolic function similar to his traditional hyperbolic function for the immunoglobulins A, G, M. Our study included a total of 168 patients with either MS according to the McDonald criteria of 2017, clinically isolated syndrome (CIS) with conversion to MS during follow-up, or stable CIS. Positive oligoclonal bands were compared with the KFLC index, Reiber’s KFLC diagram, Presslauer’s KFLC exponential curve, and Senel’s linear curve for KFLC. Reiber’s diagram detected an intrathecal production of KFLC in 98/100 patients with MS, only one patient fewer than oligoclonal bands positivity (99/100). By using the KFLC index ≥ 5.9, Presslauer’s KFLC exponential function, and Senel’s linear curve two more patients would not have been identified (96/100). For the group of patients who converted from CIS to MS similar results were obtained for both the oligoclonal bands and the Reiber graph (21/24, 88%). The KFLC index ≥ 5.9, Presslauer’s method, and Senel’s linear function each identified two patients fewer (19/24, 79%). In patients with stable CIS, 11/44 patients (25%) displayed oligoclonal bands in contrast to 9/44 patients (20%) with elevated KFLC by using Reiber’s diagram and Presslauer’s method, 8/44 patients (18%) with elevated KFLC as detected by Senel’s linear function, and 7/44 patients (16%) with KFLC index ≥ 5.9. In conclusion, Reiber’s KFLC diagram shows a great diagnostic performance to detect an intrathecal KFLC production in patients with MS.

## 1. Introduction

In a recent article Reiber and colleagues presented the concept of a quotient diagram with a hyperbolic function to establish a reference line for kappa free light chains (KFLC) in cerebrospinal fluid (CSF) [1]. Oligoclonal bands, which are the gold standard for determination of an intrathecal immunoglobulin G synthesis, were recently included in the latest McDonald criteria of 2017 to diagnose relapsing multiple sclerosis (MS) as a substitute for dissemination in time [2]. The diagnostic sensitivity of these novel criteria with a focus on oligoclonal bands was published in different cohorts in the last year [3].

Measurement of KFLC is considered to be a promising alternative to demonstrate intrathecal immunoglobulin synthesis which is technically less demanding and less expensive [4]. The CSF/serum KFLC quotient with reference to the albumin CSF/serum concentration quotient (Q_Kappa_/Q_Alb_), the so-called the KFLC index, is the most frequently used approach to determine an intrathecal synthesis of KFLC [4,5]. However, empirically defined threshold KLFC indices vary considerably and the following values were suggested: 3.6, 5.9, 6.07, and 12 [6,7,8,9]. In opposition to the non-physiological linear index, Reiber developed a theoretically and empirically founded hyperbolic function in analogy to his well-established hyperbolic evaluation schemes for immunoglobulins G, A, M aiming to improve the diagnostic sensitivity and specificity for determination of an intrathecal synthesis of KLFC [1]. Similar to Reiber graphs for immunoglobulins intrathecal synthesis can be assumed when the CSF/serum KFLC quotient (Q_Kappa_) is above the hyperbolic border line Q_Kappa_(lim) [10]. In a large study, comprising 420 patients, Presslauer and colleagues developed a formula resulting in an exponential curve and validated the method in a multicenter study later [4,8]. Senel and colleagues analyzed KFLC in an even larger cohort with 1224 patients with a different formula for the QAlb dependent upper reference value [11]. 

Here, we applied the new Reiber’s diagram to a cohort of patients who presented with MS or CIS in order to assess the real-life performance of Reiber’s KFLC diagram. In addition, we included the method proposed by Presslauer and colleagues, the method proposed by Senel and colleagues, and the KFLC index [5,8,11].

## 2. Methods

### 2.1. Patients

We retrospectively investigated paired CSF and serum samples from routine lumbar puncture from patients with MS according to the McDonald criteria of 2017 at baseline, CIS who converted to MS during follow-up, or CIS with a stable course during follow-up, who were admitted to the Department of Neurology of the Hannover Medical School from 2010 to 2018. The inclusion criteria were: (1) relapse symptoms suggestive for MS and not attributable to other autoimmune causes such as connective tissue diseases or infectious diseases that could mimic MS; (2) clinical follow-up for at least 12 months for patients who did not fulfil the McDonald criteria of 2017 for MS (follow up median: 50 months, range 13–87 months); (3) spinal tap was performed before diagnosis; (4) demographic, neuroimaging, laboratory and clinical data were available; (5) surplus paired CSF and serum samples were stored. The study was performed in accordance with local regulations and was approved by the Ethics Committee of the Hannover Medical School (No. 7837_BO_K_2018, approved on 6 April 2018). A subset of this cohort (147 patients) was previously described in studies in which the role of KFLC index and oligoclonal bands as biomarker in MS were investigated [12,13,14].

### 2.2. CSF and Serum Analytical Procedures

Paired CSF and serum samples were collected as part of routine diagnostic work-up and analyzed in the Neurochemistry Laboratory of the Department of Neurology. Samples were collected and stored at −80 °C within one day after sampling. Concentrations of albumin, IgG, IgA, and IgM were determined by kinetic nephelometry (Beckman Coulter IMMAGE) after immune complex formation using antibodies (Beckman Coulter GmbH) against albumin, IgG, IgA, and IgM. Quantitative intrathecal synthesis of IgG was calculated by the method of Reiber-Felgenhauer [10]. CSF-specific oligoclonal bands were determined by isoelectric focusing on polyacrylamide gels with consecutive silver staining [15]. The concentrations of KFLC in sera and CSF were measured by nephelometry with N Latex FLC kappa kit (Siemens Healthcare Diagnostics Products GmbH) according to the manufacturer’s instruction on a BN Prospec analyzer (Siemens Healthcare Diagnostics Products GmbH). The KFLC index was calculated by the formula (KFLC CSF/KFLC serum)/(albumin CSF/albumin serum). Values above the empirically defined threshold KLFC index ≥ 5.9 were considered as an elevated KFLC index indicating an intrathecal synthesis of KFLC [5]. For the exponential function of Presslauer, the subsequent formula was used: KFLC_IF_ = KFLC_Loc_/KFLC_CSF_ × 100 with KFLC_Loc_ = (KFLC_Ratio_ − KFCL_Lim_) × KFLC_Serum_, KFLC_Lim_ = 0.9358 × QAlb^0.6687^ and KFLC_Ratio_ = (KFLC CSF/KFLC Serum) [4]. Senel’s linear function is defined as: QKFLC = 14.85 + 2.41 × QAlb [11]. Reiber’s diagram is defined by the formula: KFLC_IF_ = KFLC_Loc_/KFLC_CSF_ × 100 or (1 − KFLC_Lim_/KFLC_Ratio_) × 100 with KFLC_Loc_ = (KFLC_Ratio_ − KFLC_Lim_) × KFLC Serum, and KFLC_Lim_ = (3.27 × (QAlb + 33) − 8.2) × 10^3^ [1]. In addition, values were inserted in the double logarithmic quotient diagram (Reibergram) [1]. The Neurochemistry Laboratory of the Department of Neurology participates regularly in the external INSTAND survey program for analytic methods quality control [16].

### 2.3. Statistical Analysis

The statistical analysis was performed with GraphPad Prism version 5.02. FLC-K Statistics and Graphic Program by Albaum IT-Solutions was used for KFLC analysis. Statistical significance in categorical data (oligoclonal bands vs. different methods) was assessed by Fisher’s exact test. Statistical significance was considered for *p*-values < 0.05.

## 3. Results

**Figure 1:**
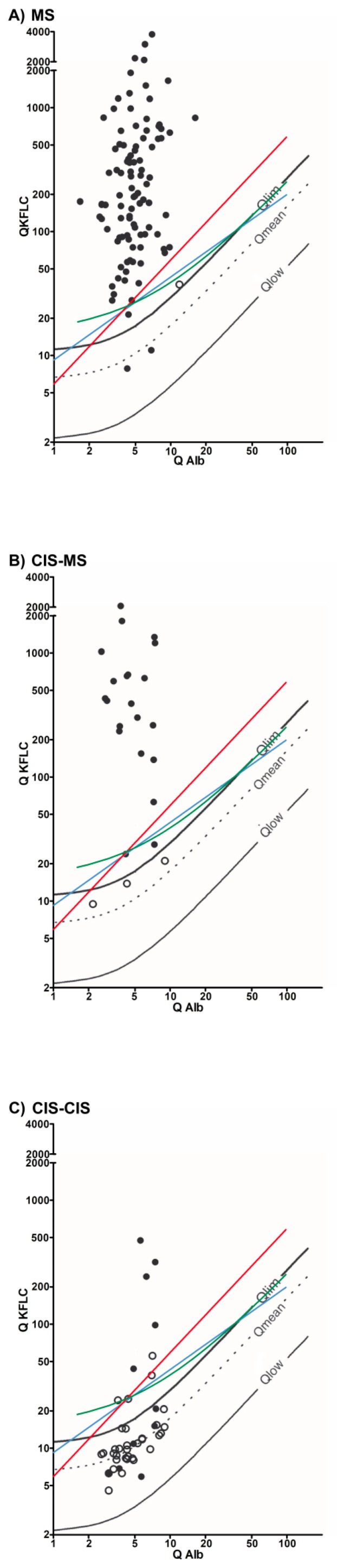
Oligoclonal bands and KFLC synthesis. The black lines represent Reiber’s diagram for KFLC, the blue lines Presslauer’s KFLC exponential curve, the green lines Senel’s linear function, and the red lines the linear KFLC index of 5.9. Black dots are patients with positive oligoclonal bands while black circles are oligoclonal band negative patients. Dots above the threshold lines represent positive patients for the used method. Depicted are patients who were diagnosed with MS according to the McDonald criteria of 2017 (**A**), CIS and conversion to MS during follow-up (**B**), and CIS with a stable disease course during follow-up (**C**).

Our study comprises a total of 168 patients with a complete MS work-up including KFLC measurements in CSF and paired serum samples. Figure 1 depicts Q_Kappa_ with corresponding Q_Alb_ for each patient. Table 1 presents an overview of the different methods to assess an intrathecal KFLC synthesis.

A very high number of 99 patients (99%) with MS according to the McDonald criteria of 2017 presented oligoclonal bands in their cerebrospinal fluid. Reiber’s KFLC diagram revealed an intrathecal KFLC synthesis in 98 patients (98%), only one patient fewer than oligoclonal bands (*p* > 0.9999). By using the KLFC index threshold of 5.9, elevated values were found in 96 patients (96%), which is three patients fewer than oligoclonal bands (*p* = 0.3687). Similarly, Presslauer’s KFLC exponential curve and Senel’s linear curve detected 96 patients (96%) indicating intrathecal KFLC synthesis (*p* = 0.3687).

24 patients with CIS converted to MS during follow-up. Of these patients, 21 patients (88%) expressed oligoclonal bands in cerebrospinal fluid at the time of the initial manifestation. Reiber’s KFLC diagram detected an intrathecal production of KFLC at the same frequency as oligoclonal bands in this group of patients (21 patients; 88%; *p* = 1.0). The KFLC index ≥ 5.9, Presslauer’s KFLC method, and Senel’s KFLC method each identified 19 patients (79%; *p* = 0.7008).

44 patients with CIS remained stable during follow-up. Of these patients, 11 patients (25%) displayed oligoclonal bands in cerebrospinal fluid. Reiber’s KFLC diagram and Presslauer’s KFLC method showed both an intrathecal synthesis of KFLC in nine patients (20%; *p* = 0.7997). Senel’s KFLC method detected eight patients (18%) with an intrathecal KFLC synthesis (*p* = 0.4526). Seven patients (16%) showed an elevated KFLC index (*p* = 0.4285).

## 4. Discussion

We compared four different methods to assess an intrathecal synthesis of KFLC in order to help standardize this promising biomarker for the diagnosis of MS. Although measurement of KFLC is easy to perform, it has not yet found its way into clinical routine [9,17]. One of the reasons might be the disagreement about the “correct” cut-off for the KFLC index, which is used to determine the intrathecal produced KFLC fraction [9,17]. In addition to the inconsistent use of KFLC index cut-off values, a CSF/serum index reflects the non-linear relationship between blood-derived molecules of different size only inaccurate [1]. Presslauer and colleagues investigated the performance of an intrathecal KFLC synthesis by using a non-linear function referring to an albumin quotient-dependent discriminative upper normal limit and confirmed its diagnostic value [4,8]. Senel and colleagues used a linear regression to establish a function for the upper normal limit of Q KFLC depending on the according albumin quotient and defined the function for a Q albumin range between 1.6 × 10^−3^ and 25.7 × 10^−3^ [11]. The diagnostic sensitivity of this linear function was investigated in different neurological diseases in the same study [11]. In a recent study, Reiber and colleagues employed a double log plot, corresponding to Reiber’s traditional diagrams to detect intrathecal synthesis of IgG, IgA and IgM, with the intention to include both rather low and high albumin quotients [1]. In our study, we compared oligoclonal bands, which are considered to represent the gold standard of intrathecal IgG production, with existing KFLC evaluation methods, either hyperbolic, exponential, or linear [1,5,8,11].

The results show a slight preponderance of oligoclonal band determination over quantitative measurements of KFLC in patients with MS. Among the different methods to calculate an intrathecal KFLC production, the highest consistency was found with Reiber’s hyperbolic curve [1]. Apart from the higher diagnostic performance of Reiber’s diagram compared to the alternative approaches to asses for an intrathecal KFLC synthesis in our cohort, Reiber’s diagram considers physiological understandings of cerebrospinal fluid flow. It is based on the laws of diffusion and has already been successfully applied to IgG, IgA, and IgM. The novel scheme for kappa light chains includes heretofore unprecedented high albumin quotients up to 150. Kappa light chains have a smaller molecular weight (22 kD) than the reference protein albumin (67 kD). Accordingly, the Reiber formula correctly predicts faster diffusion of kappa free light chains compared to the dimeric free lambda chains (45 kD) and also of albumin.

However, both methods, determination of oligoclonal bands and measuring KFLC present different strategies to detect an intrathecal immunoglobulin synthesis. Quantitative measurements of KFLC rely on a statistical evaluation, whereas the presence of oligoclonal bands compares the qualitative pattern of IgG in serum and CSF in a certain individual. We therefore suggest a sequential use of measuring KFLC and determination of oligoclonal bands as a concept of a dual assay to improve identifying patients with MS or risk to develop MS and to avoid false-positive diagnosis of MS.

In conclusion, our results imply to use Reiber’s KFLC diagram to determine intrathecal KFLC synthesis in patients with MS.

## Figures and Tables

**Table 1:** Demographic and laboratory characteristics of patients diagnosed with MS according to the McDonald criteria of 2017, CIS who converted to MS during follow up and patients with stable CIS. Laboratory characteristics include the determination of oligoclonal bands, the kappa free light chains index and the proportion of an intrathecal synthesis of KFLC by using the methods of Reiber, Presslauer, and Senel [1,8,11].

Characteristics	MS (McDonald 2017), *n* = 100	CIS to MS, *n* = 24	Stable CIS, *n* = 44
Oligoclonal bands, *n* (%)	99/100 (99%)	21/24 (88%)	11/44 (25%)
Reiber´s KFLC diagram, *n* (%)	98/100 (98%)	21/24 (88%)	9/44 (20%)
Presslauer´s KFLC curve, *n* (%)	96/100 (96%)	19/24 (79%)	9/44 (20%)
Senel´s KFLC curve, *n* (%)	96/100 (96%)	19/24 (79%)	8/44 (18%)
KLFC index > 5.9, *n* (%)	96/100 (96%)	19/24 (79%)	7/44 (16%)
Intrathecal IgG-synthesis, *n* (%)	59/100 (59%)	18/24 (75%)	2/44 (5%)
Intrathecal IgM-synthesis, *n* (%)	33/100 (33%)	4/24 (17%)	2/44 (5%)
Intrathecal IgA-synthesis, *n* (%)	11/100 (11%)	2/24 (8%)	0/44 (0%)
age, median (range)	32 (16-73)	30.5 (15-73)	36 (16-53)
females, *n* (%)	73/100 (73%)	15/24 (63%)	28/44 (64%)
